# Vital Signs: Improving Antibiotic Use Among Hospitalized Patients

**Published:** 2014-03-07

**Authors:** Scott Fridkin, James Baggs, Ryan Fagan, Shelley Magill, Lori A. Pollack, Paul Malpiedi, Rachel Slayton, Karim Khader, Michael A. Rubin, Makoto Jones, Matthew H. Samore, Ghinwa Dumyati, Elizabeth Dodds-Ashley, James Meek, Kimberly Yousey-Hindes, John Jernigan, Nadine Shehab, Rosa Herrera, L. Clifford McDonald, Amy Schneider, Arjun Srinivasan

**Affiliations:** 1 National Center for Emerging and Zoonotic Infectious Diseases, CDC; 2 University of Utah and VA Salt Lake City Health System; 3 University of Rochester Medical Center; 4 Connecticut Emerging Infections Program, Yale School of Public Health

## Abstract

**Background:**

Antibiotics are essential to effectively treat many hospitalized patients. However, when antibiotics are prescribed incorrectly, they offer little benefit to patients and potentially expose them to risks for complications, including *Clostridium difficile* infection (CDI) and antibiotic-resistant infections. Information is needed on the frequency of incorrect prescribing in hospitals and how improved prescribing will benefit patients.

**Methods:**

A national administrative database (MarketScan Hospital Drug Database) and CDC’s Emerging Infections Program (EIP) data were analyzed to assess the potential for improvement of inpatient antibiotic prescribing. Variability in days of therapy for selected antibiotics reported to the National Healthcare Safety Network (NHSN) antimicrobial use option was computed. The impact of reducing inpatient antibiotic exposure on incidence of CDI was modeled using data from two U.S. hospitals.

**Results:**

In 2010, 55.7% of patients discharged from 323 hospitals received antibiotics during their hospitalization. EIP reviewed patients’ records from 183 hospitals to describe inpatient antibiotic use; antibiotic prescribing potentially could be improved in 37.2% of the most common prescription scenarios reviewed. There were threefold differences in usage rates among 26 medical/surgical wards reporting to NHSN. Models estimate that the total direct and indirect effects from a 30% reduction in use of broad-spectrum antibiotics will result in a 26% reduction in CDI.

**Conclusions:**

Antibiotic prescribing for inpatients is common, and there is ample opportunity to improve use and patient safety by reducing incorrect antibiotic prescribing.

**Implications for Public Health:**

Hospital administrators and health-care providers can reduce potential harm and risk for antibiotic resistance by implementing formal programs to improve antibiotic prescribing in hospitals.

## Introduction

Antibiotics offer tremendous benefit to patients with infectious diseases and are commonly administered to patients cared for in U.S. hospitals. However, studies have demonstrated that treatment indication, choice of agent, or duration of therapy can be incorrect in up to 50% of the instances in which antibiotics are prescribed (1). One study reported that 30% of antibiotics received by hospitalized adult patients, outside of critical care, were unnecessary; antibiotics often were used for longer than recommended durations or for treatment of colonizing or contaminating microorganisms (2).

Incorrect prescribing of antibiotics exposes individual patients to potential complications of antibiotic therapy, without any therapeutic benefit. One such complication is infection with *Clostridium difficile*, an anaerobic, spore-forming bacillus that causes pseudomembranous colitis, manifesting as diarrhea that often recurs and can progress to sepsis and death; CDC has estimated that there are about 250,000 *C. difficile* infections (CDI) in hospitalized patients each year (3). Other complications related to unnecessary use of antibiotics include infection with antibiotic-resistant bacteria (4) and complications from adverse events (5).

Evidence is accumulating that interventions to optimize inpatient antibiotic prescribing can improve patient outcomes (6). To assist health-care providers to reduce incorrect inpatient prescribing, information is needed regarding how frequently incorrect prescribing occurs in hospitals and how improving prescribing will benefit patients. In this report, current assessments of the scope of inpatient antibiotic prescribing, the potential for optimizing prescribing, and the potential benefits to patients are described.

## Methods

The objectives of this evaluation were to 1) describe the extent and rationale for antibiotic prescribing in U.S. acute care hospitals, 2) present data illustrating the potential for improving prescribing in selected clinical scenarios, and 3) estimate the potential reductions in CDI among patients when antibiotic use is improved. For this report, antibiotics include parenteral, enteral, and inhaled antibacterial agents.

The first objective was accomplished using proprietary administrative data from the Truven Health MarketScan Hospital Drug Database (HDD) and data from CDC’s Emerging Infections Program (EIP). EIP is a network of state health departments, academic institutions, and local collaborators funded by CDC to assess the effect of emerging infections and evaluate methods for their prevention and control.* Antibiotic prescribing data and patient demographics were obtained from HDD, which contains individual billing records for all patients from a large sample of U.S. hospitals.† Antibiotic agents and doses provided were identified for all patients discharged during 2010. Age group-specific proportions of hospitalizations during which antibiotics were prescribed were calculated by antibiotic group. In 2011, EIP performed an antibiotic use prevalence survey in acute care hospitals within the 10 EIP sites. Each hospital selected a single day on which to conduct the survey on a random sample of inpatients. EIP data collectors gathered information on antibiotics given to patients and determined the rationale for antibiotic use.

For the second objective, additional data from the EIP were used to determine the frequency of opportunities to improve prescribing for selected urinary tract infections (UTIs) and prescribing of intravenous vancomycin. In addition, data reported during October 2012–June 2013 to the National Healthcare Safety Network (NHSN) Antimicrobial Use Option were analyzed; key percentile distributions of usage rates and differences in usage (between usage at 90th percentile and at 10th percentile) were calculated. This difference should be small when comparing usage rates among patient care locations caring for similar types of patients.

The third objective was accomplished through development of a dynamic model that was used to interpret the findings of an observational study and predict changes in CDI with changes in antibiotic use. First, a retrospective cohort study was conducted to quantify the relative risk for CDI using hospital discharge data and pharmacy data from two large academic centers, in New York and Connecticut, linked to active population-based CDI surveillance data from the EIP (6). The primary outcome was hospital-associated CDI (CDI >2 days after hospital admission and ≤180 days after discharge). Primary exposure of interest was receipt of inpatient broad-spectrum antibiotics (i.e., 3rd and 4th generation cephalosporins, beta-lactam/beta-lactamase inhibitor combinations, and fluoroquinolones) during hospitalization. A multivariate logistic model was used to estimate an adjusted risk ratio controlling for age, sex, Gagne comorbidity score (7), hospital, and hospital CDI rates. A stochastic, compartmental model of hospital CDI that represented distinct states of infection (uncolonized, colonized, and symptomatic) was constructed. Antibiotic use was classified with respect to type (high- and low-risk) and where the patient was in the treatment pathway (untreated, treated, and post-treatment). The model was calibrated based on the results of the epidemiologic analyses described in this report and drew other parameter estimates from stochastic distributions based on a previously published agent-based model (8).§

## Results

**FIGURE 1:**
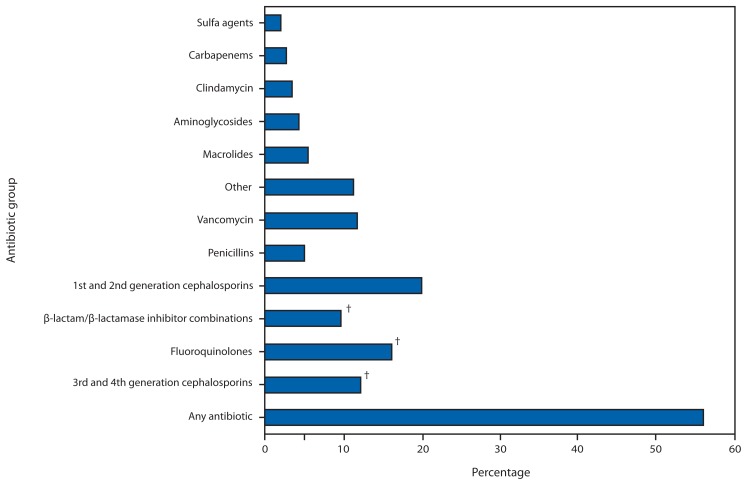
Percentage of hospital discharges with at least one antibiotic day, by antibiotic group — 323 hospitals, United States, 2010* * Data provided by Truven Health MarketScan Hospital Drug Database. ^†^ Antibiotics from these three groups, which are considered to place patients at high risk for developing *Clostridium difficile* infection, were administered to 29.8% of the patients.

In 2010, based on data obtained from all 323 hospitals by MarketScan HDD, 55.7% of patients received an antibiotic during their hospitalization, and 29.8% received at least 1 dose of broad-spectrum antibiotics (Figure 1). The EIP evaluated 11,282 patients in 183 hospitals in 2011, of whom 4,189 (37.1%) had received one or more antibiotics to treat active infections; half (49.9%) of all treatment antibiotics were prescribed for treatment in one or more of three scenarios: lower respiratory infections, UTIs, or presumed resistant Gram-positive infections (Table 1). Prescribing scenarios at a convenience sample of 36 hospitals across eight EIP sites were reviewed. Reviews of 296 instances of treatment in two specific scenarios (UTIs in patients without indwelling catheters, and treatment with intravenous vancomycin) identified that antibiotic use could potentially have been improved in 37.2% (39.6% of 111 UTI patients, 35.7% of 185 vancomycin patients); improvement opportunities mostly involved better use of diagnostic testing (Table 2).

**FIGURE 2:**
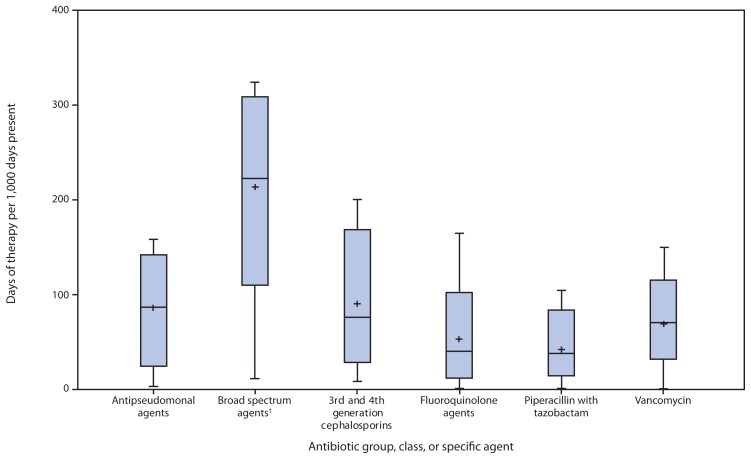
Rate of antibiotic use, by antibiotic group, class, or specific agent, among medical and surgical patients in 26 wards at 19 acute care hospitals — National Healthcare Safety Network Antimicrobial Use Option, October 2012–June 2013* * Horizontal lines represent median, 10th and 90th percentile values; whisker points are the minimum and maximum values. Plus sign is the mean value. ^†^ Including fluoroquinolones, β-lactam/β-lactamase inhibitor combinations, and 3rd and 4th generation cephalosporins.

NHSN began receiving antibiotic use data in 2012. Among the 19 hospitals reporting to the NHSN Antimicrobial Use Option that had completed data validation and submitted antibiotic use data from one or more patient care locations, results were reported for 266 patient care locations. Among the six most common types of patient locations, critical care units reported higher rates of antibiotic use (median = 937 days of therapy/1,000 days-present) compared with ward locations (median = 549 days of therapy/1,000 days-present). The variability in usage rates within any one patient location type was highest (threefold difference between 90th and 10th percentile) among combined medical/surgical wards (i.e., 26 wards categorized as caring for a mixture of medical and surgical patients). When limiting the comparisons within combined medical/surgical wards, differences in usage were eightfold for fluoroquinolones, sixfold for antipseudomonal agents, threefold for broad-spectrum agents (antibiotics considered high risk for subsequent CDI), and threefold for vancomycin (Figure 2). Overall, in the cohort study, the risk for CDI among patients unexposed and exposed to antibiotics was 6.8 and 24.9 per 1,000 discharges respectively. Multivariate modelling adjusting for covariates, for all ages combined, estimated the adjusted relative risk for development of CDI within 180 days after inpatient exposure to broad-spectrum antibiotics to be 2.9 (95% confidence interval = 2.3–3.5). The dynamic model, which accounts for both direct and indirect effects, predicted that a 30% decrease in exposure to broad-spectrum antibiotics in hospitalized adults would lead to a 26% decrease in CDI (interquartile range = 15%–38%). Such a reduction in broad-spectrum use equates to an approximately 5% reduction in the proportion of hospitalized patients receiving any antibiotic.

**Key Points**

- Antibiotics are commonly prescribed in hospitals.
- Evidence of incorrect prescribing and observed variability in current usage patterns suggest that improvements are needed and will benefit patients.
- CDC recommends that all hospitals implement antibiotic stewardship programs that include, at a minimum, seven core elements: 1) leadership support; 2) accountability through a single physician lead; 3) drug expertise through a single pharmacy lead; 4) action including at least one intervention, such as an “antibiotic timeout,” to improve prescribing; 5) tracking prescribing and resistance patterns; 6) reporting local prescribing and resistance information directly to clinicians, and 7) education for clinicians.
- Urgent action is needed to promote correct antibiotic prescribing to ensure these lifesaving drugs work in the future.
- Additional information is available at http://www.cdc.gov/vitalsigns.

## Conclusions and Comment

Antibiotics are prescribed for the majority of patients hospitalized in U.S. acute care hospitals, usually to treat infections. This post prescription review of two common prescribing scenarios for treating suspected infections identified opportunities to improve 37.2% of prescriptions, often by timely use of diagnostic tests or documentation of symptoms. This observation is similar to results of older studies (1) and a recent study (2) documenting that about 30%–50% of prescribing might be incorrect. Although the aspect of prescribing that could be improved has varied between studies, it usually involves the wrong dose or wrong duration (2). The EIP review focused on relatively objective criteria, including established standards around diagnostic testing and documentation of symptoms supporting the presence of infection. A threefold difference in overall antibiotic use in the most common patient care location, where more similar usage rates would be expected, considering similar types of patients are being cared for in these locations, is additional evidence of opportunities for improvement. This difference is a conservative measure made by comparing usage reported at the 90th percentile distribution compared with that at the 10th percentile distribution, among locations caring for similar types of patients. The magnitude of differences seen in some antibiotic groups might be the result of differences in formulary or clinical practice guidelines in place at different institutions. However, within similar location types, twofold differences were consistently measured. Although some of these differences might be attributable to differences in the mix of patients within these similar patient care locations, it is likely some might be explained by differences in prescribing practices. This type of monitoring system, which involves antibiotic use measurement to inform quality improvement activities, has been cited as an urgent need by a recent government report (10).

The data in this report confirm the findings of several previous studies demonstrating that antibiotic prescribing in hospitals is common and often incorrect. In particular, patients are often exposed to antibiotics without proper evaluation and follow-up. Misuse of antibiotics puts patients at risk for preventable health problems. These include immediate complications; antibiotics are among the most frequent causes of adverse drug events among hospitalized U.S. patients (11), and near-term complications, such as CDI, which can be severe and even deadly (9). The analysis of risk for CDI from exposure to broad-spectrum antibiotics during hospitalization found an exposed patient was at three times greater risk than a patient without this exposure. Elevated risks of similar magnitude were observed in previous studies (12,13). An estimated 30% reduction in use of these broad-spectrum antibiotics (which would reduce overall antibiotic use by only 5%) would prevent 26% of CDI related to inpatient antibiotic use. Reductions in CDI of this magnitude could also have additional positive effects in reducing transmission of *C. difficile* throughout the community.

An additional near-term complication of the unnecessary and incorrect use of inpatient antibiotics is the growing problem of antibiotic resistance in U.S. hospitals, creating treatment challenges not only for patients who are exposed to the antibiotics, but for other patients to whom these resistant bacteria spread (3). Some hospitalized patients now have infections for which there are no available antibiotic treatments (14). Urgent action is required to address this growing public health crisis. Improving the prescribing of antibiotics in hospitals is one important part of a broader strategy to counter the increase in antibiotic resistance. The CDC report, Antibiotic Threats in the United States, 2013, addresses other priority needs to reduce antibiotic resistance, including preventing infections and the spread of resistance, tracking resistance patterns, and developing new antibiotics and diagnostic tests (3).

Programs dedicated to improving antibiotic prescribing in hospitals are commonly referred to as antibiotic stewardship programs. Such programs serve to ensure optimal treatment for hospitalized patients with infection and reduce unnecessary antibiotic use to minimize harm to patients and prolong the length of time antibiotics are effective (15). Variability in the types of patients and available resources and expertise between hospitals calls for flexibility in how these programs are implemented. However, experience demonstrates that these programs can be successful in a wide variety of hospital types to reduce overall and incorrect antibiotic prescribing, decrease drug costs, prevent adverse events caused by antibiotics, and reduce CDI rates and antibiotic resistance locally (6,15). Although cost savings from these programs will vary depending on the size of the facility and the extent to which interventions are implemented, published studies from mostly larger settings have consistently shown significant annual savings ($200,000–$900,000) (1).

Correct antibiotic treatment (e.g., prompt treatment of sepsis) is critical to saving lives of hospitalized patients with certain infectious diseases. Given the proven benefit of hospital stewardship programs to patients and the urgent need to address the growing problem of antibiotic resistance, CDC recommends that all hospitals implement an antibiotic stewardship program. CDC has developed guidance that can assist hospitals in either starting or expanding a program to improve antibiotic prescribing (16). Central to this guidance are seven core elements that have been critical to the success of hospital antibiotic stewardship programs (Box). In addition to highlighting these key elements for success of stewardship programs, the CDC guidance also provides background information on the proven benefits of improving antibiotic prescribing in hospitals and more details on the structural and functional aspects of successful programs. To accompany the guidance, CDC also has developed a stewardship assessment tool that includes a checklist to help facilities assess the status of their efforts to improve antibiotic prescribing and point out potential areas for further improvement (16).

BOX **Seven core elements critical to the success of hospital antibiotic stewardship programs**

- Leadership commitment: Dedicating necessary human, financial, and information technology resources.
- Accountability: Appointing a single leader responsible for program outcomes. Experience with successful programs has shown that a physician leader is effective.
- Drug expertise: Appointing a single pharmacist leader responsible for working to improve antibiotic use.
- Action: Implementing at least one recommended action, such as systemic evaluation of ongoing treatment need after a set period of initial treatment (i.e., “antibiotic time out” after 48 hours).
- Tracking: Monitoring antibiotic prescribing and resistance patterns.
- Reporting: Regular reporting information on antibiotic use and resistance to doctors, nurses and relevant staff members.
- Education: Educating clinicians about resistance and optimal prescribing.

**Source:** CDC. Core elements of hospital antibiotic stewardship programs. Atlanta, GA: US Department of Health and Human Services, CDC; 2014. Available at http://www.cdc.gov/getsmart/healthcare/implementation/core-elements.html.

## Figures and Tables

**TABLE 1:** Prevalence of antibiotic use among randomly selected patients in 183 acute care hospitals — Emerging Infections Program health-care–associated infections and antimicrobial use prevalence survey, United States, 2011

Antibiotic use assessment	No.	(%)
**Total no. of patients in the survey**	**11,282**	**—**
Patients on any antibiotic to treat an active infection	4,189	(37.1)
Treatment indication for antibiotic*	7,199	—
For LRI (community onset), with or without BSI	1,596	(22.2)
For UTI (health-care or community onset), with or without BSI	993	(13.8)
For presumptive resistant Gram-positive infection treated with vancomycin (intravenous), linezolid, or daptomycin	1,270	(17.6)
No. of antibiotics with one or more treatment indications above	3,592	(49.9)

**Abbreviations:** LRI = lower respiratory tract infection; BSI = bloodstream infection; UTI = urinary tract infection.

* Indications are not mutually exclusive.

**TABLE 2:** Assessment of antibiotic prescribing among inpatients in 36 hospitals treated for urinary tract infection (UTI) without indwelling catheter or treated with intravenous vancomycin — Emerging Infections Program health-care–associated infections and antimicrobial use prevalence survey, United States, 2011

Treatment	No.	(%)
**Patients treated for UTI present on admission, without indwelling catheter**	**111**	**—**
Urine culture was not ordered, although standard practice before treatment	18	(16.2)
Urine culture was positive, but no documented symptoms were present	23	(20.7)
Urine culture was negative, and no documented symptoms were present	3	(2.7)
No. of patients with potential for improvement in prescribing	44	(39.6)
**Patients treated with intravenous vancomycin**	**185**	—
No diagnostic culture obtained around antibiotic initiation, although standard practice with most infections	17	(9.2)
Diagnostic culture showed no Gram-positive bacterial growth, but patient still treated for long duration (>3 days) (excludes presumed SSTI, which often can be culture negative)	40	(21.6)
Diagnostic culture grew only oxacillin-susceptible *Staphylococcus aureus*, but patient still treated for long duration (>3 days) (likely missed opportunity to switch antibiotic based on culture result)	9	(4.9)
No. of patients with potential for improvement in prescribing	66	(35.7)
**Combined UTI or vancomycin prescribing**	**296**	—
Total no. of patients with potential for improvement in prescribing	110	(37.2)

**Abbreviation:** SSTI = skin and soft tissue infection.

## Footnotes

* Additional information available at http://www.cdc.gov/ncezid/dpei/eip/index.html.

† A proprietary system integrating data systems from claims and hospital-based data systems among a convenience sample of hospitals and providers. Additional information available at http://truvenhealth.com.

§ Additional information available at http://www.cdc.gov/getsmart/healthcare/evidence/cdiff.html.
